# Evaluation of role of Tigecycline among clinically significant multidrug resistant pathogens from a tertiary care hospital

**DOI:** 10.12688/f1000research.141535.2

**Published:** 2024-06-06

**Authors:** Annapoorna Remash, Pooja Rao, Suchitra Shenoy, Shrikala Baliga, Shafir Kassim

**Affiliations:** 1 Microbiology, School of Health Sciences, Kannur University, Talassery, Kerala, India; 2 Microbiology, Kasturba Medical College, Mangalore, Manipal Academy of Higher Education, Manipal, Karnataka, 576104, India; 3 Internal Medicine, Aster clinic, Sharjah, United Arab Emirates

**Keywords:** Tigecycline, MRSA treatment, Extended Spectrum Beta Lactamases, Multi Drug Resistant treatment

## Abstract

**Background:**

Tigecycline, a glycylcycline antibiotic is a promising option for the treatment of single or multidrug resistant pathogens. The aim of the study was to evaluate the in-vitro Tigecycline susceptibility of various pathogens from clinical samples received at the tertiary care hospitals in South India.

**Methods:**

The analysis of specimens from patients admitted were carried out in this prospective cross sectional study. The identification and antimicrobial susceptibility testing was performed by semi-automated Vitek 2 systems and Kirby Bauer method. Pattern of data analysis was done by descriptive statistics.

**Results:**

Among 2574 isolates, 812 isolates were Gram positive pathogens and 1762 isolates were Gram negative pathogens. Resistance to Tigecycline was more common among Gram negative pathogens (18.62%) in comparison to the Gram positive pathogens (0.49%). Among 740 Extended Spectrum Beta Lactamases (ESBL) producers such as *Klebsiella* species & *E coli*, 629 isolates were susceptible, and 93 isolates were resistant to the tigecycline. All the methicillin resistant *Staphylococcus aureus* (MRSA) isolates were susceptible to tigecycline.

**Conclusion:**

Multidrug resistant (MDR) pathogens like *Acinetobacter* species, and *Klebsiella* species were found to be highly effective *in vitro* to tigecycline for elimination of infections caused by both Gram positive and Gram negative pathogens. The use of combination therapy becomes crucial to prevent the development of Pan Drug resistance.

## Introduction

Tigecycline is the first novel broad spectrum glycylcycline antibiotic. It belongs to the class of protein synthesis inhibitor and is bacteriostatic in its action. ^1^ Although tigecycline is structurally related to minocycline, alterations to the molecule resulted in its expanded spectrum of activity and decreased susceptibility to the development of resistance when compared to other tetracycline antibiotics. ^2^ They are administered intravenously. The vast increase in the rate of antibiotic resistant bacteria such as *Staphylococcus aureus, Acinetobacter baumannii, and Escherichia coli* has led to the development of tigecycline. ^2^ Being a tetracycline derivative the therapeutic activity of tigecycline has been expanded to include both Gram positive and Gram negative organisms including multidrug resistant organisms. ^1^^–^^3^ It exhibits strong *in vitro* activity against Gram positive, Gram negative, aerobic, anaerobic and atypical bacterial species including antibiotic resistant strains. ^4^ Due to the limited therapeutic options, the treatment of life threatening infections caused by multidrug resistant pathogens becomes a challenge. ^4^^–^^6^ The drug has its niche in therapy of pan drug resistant (PDR) organisms due to minimum drug-drug interactions, organ toxicity, handy twice daily dosing and absence of monitoring renal functions tests. ^7^

Resistance-nodulation-cell division (RND)-type transporters and other efflux pump systems has been observed as mechanism for development of resistance in *E. coli* and *Klebsiella* spp. ^8^ In this retrospective study, we analyzed the antibiotic susceptibility pattern of all the clinically significant Gram positive and Gram negative aerobic bacteria towards tigecycline. The study was designed to warrant the surveillance of tigecycline susceptibility among diverse species of bacteria from the clinical samples.

## Methods

This was a retrospective study conducted at Department of Microbiology, Kasturba Medical College Hospitals, Mangalore. The data of all clinically significant isolates from specimen such as pus, wound tissue, respiratory samples, blood and body fluids from consecutive patients over a period of one year from January 2021 to December 2021 were included in the study. Urine samples have been excluded from the study due to poor drug urine concentration of tigecycline.

The study began after the approval from Institutional Ethics Committee (IEC), KMC, Mangaluru (Reg No: IEC KMC MLR02-2020/107). The IEC has waved off the patient informed consent as it is a retrospective lab based study and also granted permission to share the data in an open repository system. The study is in agreement with Helsinki declaration, and Laboratory Information System (LIS) and microbiology records were accessed with written permission from the microbiology department incharge.

The comprehensive data which includes 2574 clinically significant isolates were retrieved from readily available data in the Laboratory Information System and microbiology records and analyzed during a two month period. The data information was collected from medical records and the experiments which were already conducted in the laboratory. The antibiotic susceptibility pattern data records which were carried out by The VITEK ^®^ 2 Compact (biomerieux, USA), a semi-automated method and Kirby-Baurer disk diffusion method and interpreted as sensitive, intermediate and resistant as per the European Committee on Antimicrobial Susceptibility Testing (EUCAST) and Food and drug administration guidelines respectively were compiled and analyzed. EUCAST criteria with the interpretation of minimum inhibitory concentration (MIC) which ranged from 0.06 to 64 μg /ml where MIC of tigecycline for Enterobacteriaciae <2 μg /ml is sensitive and >8 μg/ml was considered resistant. ^9^ For the Kirby-Baurer disk diffusion method with Tigecycline disk 15 μg (Hi-Media, India), the isolates with >19 mm zone diameter were considered sensitive while <14 mm as resistant strains. ^10^ Data records of all the *Staphylococcus aureus* isolates for Methicillin resistance using the cefoxitin disk method or by Vitek 2 compact system and Gram negative Enterobacterales data for ESBL production using ceftazidime and ceftazidime clavulunic acid or Vitek 2 compact systems were analysed. Descriptive statistics data analysis was done by entering the data into an Excel version 2308 (RRID:SCR_016137) sheet and analyzed using IBM SPSS (RRID:SCR_002865) version 25. The continuous and categorical variables have been represented as mean ± standard deviation and frequency percentages respectively.

## Results

Out of 2574 patient’s data analyzed, 64% were males and 36% were females. The tigecycline susceptibility was similar among both of these groups approximating to 85%. Resistance to tigecycline was more common among males (13.58%) than females (11.67%) where the difference is not significant.

**Figure 1.**
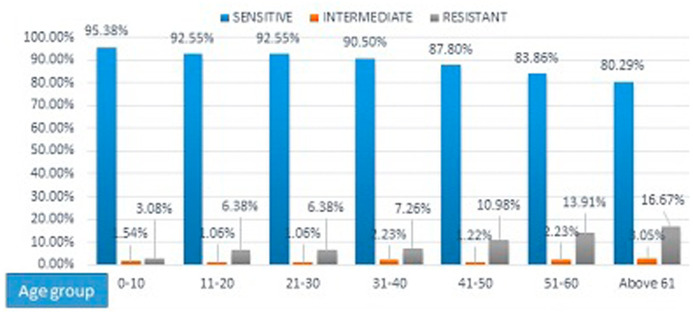
Tigecycline susceptibility among different age group.

Isolates from children in the age group 0-10 were more susceptible to tigecycline (95.38%), as the age increases there is trend for decreasing susceptibility to tigecycline ( Figure 1). Among the samples, resistance to tigecycline was most common in isolates from cerebrospinal fluid (CSF) samples (44.44%) and high susceptibility was seen in blood, body fluids (93%) and pus (91%) ( Table 1). ^11^

**Table 1.** Tigecycline susceptibility pattern among various clinical samples.

Sample	Total no.	Tigecycline susceptibility & percentage						
		%	I	%	R	%	S	%
Aspirates	61	2.4	4	6.6	10	16.4	47	77.0
Bile	29	1.1	0	0.0	6	20.7	23	79.3
BLOOD	594	23.1	7	1.2	32	5.4	555	93.4
Broncho Alveolar Lavage	43	1.7	1	2.3	9	20.9	33	76.7
CSF	9	0.3	1	11.1	4	44.4	4	44.4
Fluid	53	2.1	1	1.9	2	3.8	50	94.3
Pus	386	15.0	1	0.3	30	7.8	354	91.7
Secretions	16	0.6	1	6.3	3	18.8	12	75.0
Sputum	288	11.2	7	2.4	47	16.3	233	80.9
Swab	584	22.7	16	2.7	87	14.9	481	82.4
Endotracheal aspirate	222	8.6	16	7.2	37	16.7	168	75.7
Tissue	266	10.3	3	1.1	61	22.9	202	75.9
Tracheostomy	23	0.9	1	4.3	4	17.4	18	78.3
**Grand Total**	**2574**		**59**		**332**		**2180**	

Tigecycline demonstrates potent *in vitro* activity against most relevant pathogens. The Gram negatives and Gram positives such as *E. coli* (n=471, 99.5%) and *S. aureus* (n=480, 100%) displayed potent activity to tigecyclines respectively followed by *Enterococcus* spp. (n=77, 98.7%), *Acinetobacter* spp. (n=296, 88%) and *Klebsiella* spp. (n=401, 74.5%) with exceptions to *Pseudomonas* species & *Proteus* species ( Table 2). ^11^

**Table 2.** Tigecycline susceptibility pattern among various clinical isolates.

Organism	Number of data analyzed		Tigecycline susceptibility & percentage					
	Nos	%	S	%	I	%	R	%
*Achromobacter xylosoxidans*	3	0.12	1	33.33	0	0.00	2	66.67
*Acinetobacter species*	336	13.05	296	88.10	28	8.33	12	3.57
*Aeromonas hydrophila*	12	0.47	12	100.00	0	0.00	0	0.00
*Brevundimonas diminuta*	1	0.04	1	100.00	0	0.00	0	0.00
*Burkholderia cepacia*	11	0.43	4	36.36	2	18.18	5	45.45
*Chrysobacterium indologens*	1	0.04	1	100.00	0	0.00	0	0.00
*Citrobacter species*	34	1.32	30	88.24	2	5.88	2	5.88
CONS	162	6.29	162	100.00	0	0.00	0	0.00
*Edwardsiella tarda*	1	0.04	1	100.00	0	0.00	0	0.00
*Enterobacter species*	129	5.01	110	85.27	0	0.00	19	14.73
*Enterococcus species*	78	3.03	77	98.72	0	0.00	1	1.28
*Escherichia coli*	473	18.38	471	99.58	0	0.00	2	0.42
*Klebsiella species*	538	20.90	401	74.54	19	3.53	118	21.93
*Leclerciaade carboxylata*	2	0.08	1	50.00	1	50.00	0	0.00
Non fermenting Gram negative bacilli	1	0.04	1	100.00	0	0.00	0	0.00
*Pantoea species*	6	0.19	5	83.33	0	0.00	1	16.67
*Raoultella ornithinolytica*	1	0.04	1	100.00	0	0.00	0	0.00
*Serratia marcescens*	17	0.66	13	76.47	0	0.00	4	23.53
*Sphingomonas paucimobilis*	4	0.16	2	50.00	2	50.00	0	0.00
*Staphylococcus aureus*	481	18.69	481	100	0	0.00	0	0.0
*Stenotrophomonas maltophilia*	18	0.70	7	38.89	1	5.56	10	55.56
*Streptococcus species*	91	3.54	89	97.80	0	0.00	2	2.20
Total	2400		2166		55		179	

Amongst 740 ESBL producers, which includes 368 *Klebsiell* species and 363 *Escherichia coli,* 99 *.*72% of ESBL producing *E.coli* had higher susceptibility to tigecycline. Resistance to tigecycline was more common among ESBL *Klebsiella* species (25%), than in *E.coli* (0.28%). In 481 isolates of *Staphylococcus aureus*, 212 isolates were methicillin resistant *Staphylococcus aureus* (MRSA) and 269 isolates were methicillin sensitive *Staphylococcus aureus* (MSSA). All of the 212 MRSA isolates (100%) were susceptible to tigecycline. ^11^

Susceptibility to tigecycline was higher in Gram positive (99.5%) with MIC (≤ 0.12 μg) in comparison to Gram negative pathogens (78%) ( Table 3). The MIC for tigecycline ranged from ≤ 0.12 to 2 μg/ml for 91% of isolates which were in the susceptible range, while 8% Gram negatives isolates had MIC ≥ 8 μg which were resistant and among them 3% of them were *Klebsiella* species. ^11^

**Table 3.** Tigecyline susceptibility pattern among Gram positive and Gram negative pathogens.

Organism	Number of data analyzed		Tigecycline susceptibility & percentage					
	No.	%	S	%	I	%	R	%
Gram Positive	838	31.55%	808	99.51%	0	0.00%	4	0.49%
Gram Negative	1562	65%	1234	78.04%	59	3.35%	328	18.62%
Total	2400		2042		59		332	

## Discussion

Tigecycline is an effective alternative drug of choice for the treatment of infections caused by MDR Gram negative pathogens and MRSA. ^12^ The susceptibility trend changed as the age increased probably due to use of antimicrobial agents and previous exposure over the years to antibiotics such as Carbepenems. ^13^ In our study, out of 481 *Staphylococcus aureus* isolates all (100%) were susceptible to tigecycline. Among these 212 (44.07%) isolates were MRSA which has shown 100% susceptibility to tigecycline. This is consistent with studies conducted by Bijayini Behera *et al.,* in an Indian tertiary care hospital. ^12^ In their study, 21 MRSA isolates were analyzed to detect tigecycline susceptibility pattern. All the 21 isolates were suscetible to tigecycline. In a study conducted by Manisha *et al.*, to analyze tigecycline susceptibility pattern among MDR bacteria in a tertiary care hospital, 35 MRSA isolates were subjected to assay of which all isolates had shown susceptibility to tigecycline which is also consistent with the results from this study. ^14^ Hence, this indicates that tigecycline can be used as a therapeutic alternative for treating infections caused by MRSA and ESBL producing MDR pathogens especially in intraabdominal skin & soft tissue infections where the penetration of the drug is high at these sites when compared to blood and respiratory tract. Though the susceptibility is high in blood but the usage of tigecycline is questionable as the volume of distribution of the drug in blood is low. ^10^

The most common antibiotic resistance mechanism evolving among the family Enterobacterales is through the development of ESBL production. ^15^ In our study, the 538 Klebsiella isolates which includes 476 (88.48%) *Klebsiella pneumoniae*, 6 (1.12%) *Klebsiella oxytoca* and 56 (10.40%) isolates were identified as *Klebsiella* spp. Among the Klebsiella *pneumoniae* isolates, 350 (73.53%) were found to be susceptible to tigecycline, 13 (2.73%) isolates were found to show intermediate sensitivity and 113 (23.74%) isolates were resistant to tigecycline. Anand Manoharan *et al.,* conducted a study to evaluate tigecycline activity in clinical isolates among Indian medical centers in which 120 *Klebsiella* spp. were analyzed. ^16^ In their study all the isolates were found to be susceptible to tigecycline which is not consistent with our study. In studies conducted by Simit Kumar *et al.,* 100%, Bijayini Behera *et al.,* 97% and Soham Gupta *et al.,* 85.7%, Kusuma GR *et al.,* 97.14% sensitivity towards tigecycline while reduced susceptibility was noted in this study. ^12^^,^^17^^–^^19^ Over a decade, reduced susceptibility towards tigecycline has been noted. It was also noted that in a study performed by Subhash C Arya *et al* 66% isolates were found to be susceptible to tigecycline. ^20^

Among the Enterobacterales, *Klebsiella* species showed higher resistance with MIC>2 ug/ml in 25% of the isolates. In studies conducted by Nandi P *et al.,* 21 isolates were ESBL producers out of which only 1 (4.76%) was resistant to tigecycline which is not consistent with the present study. ^2^

In our study, out of the 473 *E. coli* isolates, 471 showed good susceptibility to tigecycline with 99.58% being sensitive and having MIC between ≤0.5 to 2 μg/ml. The data of higher *E. coli* susceptibility than *Klebsiella* spp. was similar in another study. ^20^ Out of these, 363 (76.74%) isolates were ESBL producers of which only 1 (0.28%) isolate was resistant to tigecycline and 362 (99.72%) isolates were found to be sensitive to tigecycline. Tigecycline has decreased *in vitro* activity or intrinsically resistant to *Pseudomonas* spp., *Morganella* spp., *Proteus* spp. and *Providencia* spp. ^21^ In a study conducted in Vietnam, 19 MDR Klebsiella isolates showed good susceptibility to tigecycline, colistin followed by Fosfomycin. ^22^ In another study by Ku et al, found tigecycline, fosfomycin, colistin and carbapenems demonstrated low resistance rates when tested against the ESBL-producing isolates. ^23^

## Conclusions

Tigecycline is a newly introduced antibiotic used mainly in the treatment of infections caused by multidrug resistant organisms. Tigecycline is active against the most frequently encountered pathogens including *Klebsiella* species *, E.coli, Staphylococcus aureus, Acinetobacter* species, CONS, *Enterobacter* species and *Streptococcus* species. Tigecycline has shown excellent *in vitro* activity against ESBL producing pathogens and MRSA isolates. The usage of tigecycline should be monitored routinely so as to track the development of resistance. It should be used as a reserve antibiotic to treat life threatening infections as a combination regimen with other antibiotics and also in infections caused by MDR bacteria.

## Data Availability

Dryad: Tigecycline among clinically significant multidrug resistant pathogens. https://doi.org/10.5061/dryad.sqv9s4n6z. ^11^ This project contains the following underlying data:
-
Dryad_Data_Collection.xlsx Dryad: Tigecycline among clinically significant multidrug resistant pathogens. https://doi.org/10.5061/dryad.sqv9s4n6z. ^11^ This project contains the following underlying data:
- README_file.txt.txt (The file includes the title with the authors who have made contributions to the study, duration, location, funding and sharing and access information) Data are available under a CC0 1.0 Universal (CC0 1.0) Public Domain Dedication license.
