# Supplementary material for: Evaluation of role of Tigecycline among clinically significant multidrug resistant pathogens from a tertiary care hospital
Source: F1000Res. 2024 Jun 6;13:36. Originally published 2024 Jan 8. [Version 2] doi: 10.12688/f1000research.141535.2 (PMC11170064; doi:10.12688/f1000research.141535.2)
Supplement: Peer review history

# Reviewer report: Reviewer response for version 1

**DOI:** 10.5256/f1000research.154991.r252069

**Published:** 2024-06-12

**Authors:** Richa Sinha

**Affiliations:** 1 Sanjay Gandhi Post Graduate Institute of Medical Sciences, Lucknow, Uttar Pradesh, India

This article highlights today's most common problem of Multi-drug resistant bugs. As Tigecycline is active against both Gram positive and Gram negative bacteria's, it's importance lies in it's use against MRSA, VRE and ESBLs. Though antibiogram is given for various isolates recovered and identified from the clinical samples, in the result section, the table does not highlight the percentage of staphylococcus which were identified to be MRSA, same with VRE and ESBLs.

 The authors are requested to represent the same in tabulated form.

Is the work clearly and accurately presented and does it cite the current literature?

Yes

If applicable, is the statistical analysis and its interpretation appropriate?

Yes

Are all the source data underlying the results available to ensure full reproducibility?

Yes

Is the study design appropriate and is the work technically sound?

Yes

Are the conclusions drawn adequately supported by the results?

Yes

Are sufficient details of methods and analysis provided to allow replication by others?

Yes

Reviewer Expertise:

Diagnostics, Infectious disease, Hospital surveillance

I confirm that I have read this submission and believe that I have an appropriate level of expertise to confirm that it is of an acceptable scientific standard.

# Reviewer report: Reviewer response for version 1

**DOI:** 10.5256/f1000research.154991.r275521

**Published:** 2024-05-10

**Authors:** Gamal Wareth

**Affiliations:** 1 Jena University Hospital, Fredrich-Loeffler-Institut (FLI), Jena, Germany

The article by Remash et al. stands out for its unique approach to evaluating the role of Tigecycline among clinically significant MDR pathogens from a tertiary care hospital in India. The article included a large number of pathogens collected from different clinical samples. It is well-organized and suitable for publication in this journal. However, a few minor comments are necessary before publication.

- Please change the gram to Gram all over the text. The manuscript needs little English editing
- Do you have an explanation for why isolates from children in the age group 0-10 were more susceptible to tigecycline? Please discuss this point.
- Resistance to Tigecycline is uncommon as it is a new drug and not widely used in developing countries. Several studies showed that even MDR pathogens were susceptible, for example, antibiotic susceptibility testing of the 19 K. pneumoniae strains from patients in Vietnam revealed that all isolates were susceptible to Tigecycline despite they were MDR; Please discuss this paper in discussion ( https://doi.org/10.51585/gjm.2024.1.0030)ref 1
- , please include more studies from other countries

Is the work clearly and accurately presented and does it cite the current literature?

Yes

If applicable, is the statistical analysis and its interpretation appropriate?

I cannot comment. A qualified statistician is required.

Are all the source data underlying the results available to ensure full reproducibility?

Yes

Is the study design appropriate and is the work technically sound?

Yes

Are the conclusions drawn adequately supported by the results?

Yes

Are sufficient details of methods and analysis provided to allow replication by others?

Yes

Reviewer Expertise:

AMR

I confirm that I have read this submission and believe that I have an appropriate level of expertise to confirm that it is of an acceptable scientific standard.

## References

[1] : WGS analysis of hypervirulent and MDR Klebsiella pneumoniae from Vietnam reveales an inverse relationship between resistome and virulome. *German Journal of Microbiology* .2024;
